# Peridynamics Model with Surface Correction Near Insulated Cracks for Transient Heat Conduction in Functionally Graded Materials

**DOI:** 10.3390/ma13061340

**Published:** 2020-03-15

**Authors:** Yang Tan, Qiwen Liu, Lianmeng Zhang, Lisheng Liu, Xin Lai

**Affiliations:** 1 Hubei Key Laboratory of Theory and Application of Advanced Materials Mechanics, Wuhan University of Technology, Wuhan 430070, China; tanyang@whut.edu.cn (Y.T.); Liulish@whut.edu.cn (L.L.); laixin@whut.edu.cn (X.L.); 2 Department of engineering structure and mechanics, Wuhan University of Technology, Wuhan 430070, China; 3 State Key Laboratory of Advanced Technology for Materials Synthesis and Processing, Wuhan University of Technology, Wuhan 430070, China; lmzhang@whut.edu.cn

**Keywords:** transient heat conduction, functionally graded materials, insulated crack, peridynamics, surface correction

## Abstract

A peridynamic (PD) model of functionally graded materials (FGMs) is presented to simulate transient heat conduction in the FGM plate with insulated cracks. The surface correction is considered in the model to reduce the surface effect near the domain boundary and insulated cracks. In order to verify the proposed model, a numerical example for the FGM plate is carried out. The results show good agreement with the analytical solution. The convergence of the model with the surface correction for FGMs without cracks is then investigated. The results reveal that our model converges to the classical solutions in the limit of the horizon going to zero. The effects of two material points discretization schemes on the accuracy of numerical results are investigated. For transient heat conduction of FGMs with a static crack, the results obtained from the proposed PD model agree well with that from the finite element method. Finally, transient heat conduction of the FGM plate with a dynamic horizontal crack and intersecting cracks is simulated and discussed.

## 1. Introduction

Owing to their excellent mechanical and thermal properties, FGMs have attracted great interest in the field of materials and engineering [1]. The physical properties of FGMs are non-homogeneous and the material parameters present a continuously gradient change in the direction of thickness. Compared with traditional composite materials, FGMs have the advantage of no interfacial stress. In general, due to their superior physical properties, FGMs are used as structural components in aerospace and nuclear reactors in extreme high-temperature environments. Hence, transient heat conduction analysis is important for the design, optimization and engineering applications of FGMs [2].

Heat conduction of FGMs has been investigated by many researchers, and they mainly used analytical and numerical methods. Hosseini et al. [3] studied the transient thermal conduction of a functionally graded cylindrical shell assumed to be an axisymmetric boundary condition by using the Bessel functions. Zhao et al. [4] used perturbation method to calculate the transient temperature field of a functionally gradient ceramic plate under a convective boundary condition. Kayhani et al. [5] presented an exact analytical solution for the steady state heat conduction problem of cylindrical composite laminates. The solution can be directly applied to cylindrical composite pipes and reservoirs. Cinefra et al. [6,7] proposed models for the thermo-mechanical analysis of FGM shells and FEM thermal analysis of laminated shells by referring to Carrera’s Unified Formulation (CUF).

Since the non-homogeneous material properties, it is very difficult to obtain the analytical solution of transient heat conduction equation for FGMs. Therefore, numerical methods are widely used in the heat conduction problem of FGMs. Wang et al. [8] simulated temperature field and associated thermal stresses in FGMs with temperature-dependent material properties by a finite element/finite difference (FE/FD) method. Liu and Ming [9] developed a new three-dimensional control volume finite element method (CVFEM) for transient heat conduction in FGMs. Li et al. [2] used a multiple reciprocity boundary face method to solve the transient heat conduction problem of FGMs. Yu et al. [10] solved the transient heat conduction problem of FGMs by a new method consisting of differential transformation method and radial integral boundary element method. Zhang et al. [11] solved the two-dimensional transient heat conduction problems in FGMs by the numerical manifold method (NMM). Zhou et al. [12] employed the meshless weighted least-square (MWLS) method to solve the heat conduction problem for irregular FGMs with temperature-dependent material properties. Xi et al. [13] proposed a semi-analytical boundary collocation solver for inverse Cauchy problems in heat conduction under 3D FGMs with a heat source. Krahulec et al. [14] used the meshless local radial basis function method to simulate stationary and transient heat conduction problems in FGMs.

Under extreme thermal shock conditions, crack propagation is easily induced inside the FGM [15]. The inherent difficulty of this problem is that the spatial derivatives required for partial differential equations do not exist on a crack tip or surface. Therefore, any numerical method derived from these equations inherits this difficulty in modeling cracks [16]. The PD method is a nonlocal formulation of continuum mechanics, firstly proposed by Silling [17]. This theory discretizes the continuum into material points and assumes that the material points interact with each other in a finite range. Based on the level of interaction between the two points, damage is incorporated into the theory, so localization and fracture occur naturally [16]. After nearly 20 years of development, the peridynamic theory has been widely accepted to simulate and predict the damage and fracture of materials and structures [18,19,20,21,22].

A peridynamic approach to heat conduction has advantages over other continuum theory-based methods, as it not only accounts for nonlocality but it also allows for the determination of the temperature field including the discontinuities. In the context of peridynamic theory, Bobaru and Duangpanya [23] proposed 1-D transient heat conduction. They examined the effects of constant and triangular micro-conductivity functions on the heat transfer. Actually, they attributed micro-conductivity as a feature inherited in the peridynamic model of heat conduction. They extended their work to 2-D space later, and numerically proved that when the nonlocal parameter in the peridynamic model goes to zero, the results are converged to the classical solution. The numerical calculation considered the self-transfer and volume correction of the material points. The temperature load was applied to the material point closest to the boundary, and a two-dimensional transient heat conduction problem with adiabatic cracks was simulated [24]. Agwai [25] gave three different PD heat transfer kernel functions, and the corresponding constant micro-conductivity is obtained by equalizing the thermal potential energy function. The 1-D numerical calculation shown that the surface correction factor reduces the PD numerical calculation error. Based on the Lagrangian formula and PD theory, Oterkus et al. [26] developed the state-based PD heat conduction equation. They presented a thermal response function, a method for determining the micro-conductivity and the method of the virtual layer temperature boundary condition, but their examples still using the bond-based formulation. In another work, Chen and Bobaru [27] focused on the convergence issue regarding numerical solution of the peridynamic heat transfer equation in 1-D. They employed a one-point Gauss quadrature method to solve the equation numerically and showed that type of the peridynamic kernel affects the convergence. Shojaei et al. [28] developed a simple switching technique to couple PD with methods based on the classical local theory near the boundaries to remove the surface effect from the Peridynamic solution. Bazazzadeh et al. [29] used adaptive grid refinement techniques close to the boundaries to reduce the surface effect for thermal shock problems in ceramics.

The PD heat conduction method does not include spatial derivatives and uses instead an integral over a region around a material point. The new model is suitable for modeling, for example, heat flow in bodies with evolving discontinuities such as growing insulated cracks. However, the effect of the surface correction factor on the accuracy and convergence of numerical calculation results is still unclear, especially for 2-D transient heat conduction problem. For the problem of heat conduction with a crack, no one has considered the surface correction near the crack.

To address this concern, the aim of this paper is focused to analyze the effect of surface correction on transient heat conduction of the FGM plates. This work is organized into four sections. In Section 2, we review the peridynamic formulation for transient heat conduction and describe the PD heat transfer kernel function formulas with constant and conical micro-conductivity. In Section 3, based on the kernel function of constant micro-conductivity, a discrete model for transient heat conduction in FGMs with cracks is given. Near a domain boundary and insulated cracks, we propose a surface correction factor to reduce surface effect. In Section 4, the effects of surface correction on the accuracy of PD transient thermal conduction model of FGMs are studied. Moreover, the convergence of the FGM model after surface correction is analysed and the effects of two material points discretization schemes on the accuracy of numerical results are investigated. In Section 5, the transient heat conduction problem of FGMs with static and dynamic cracks are simulated. The influence of the surface correction about the material points close to an insulated crack on the numerical results is analysed. In the last section, some conclusions are presented.

## 2. Bond-Based Peridynamic Thermal Transfer

**Figure 1:**
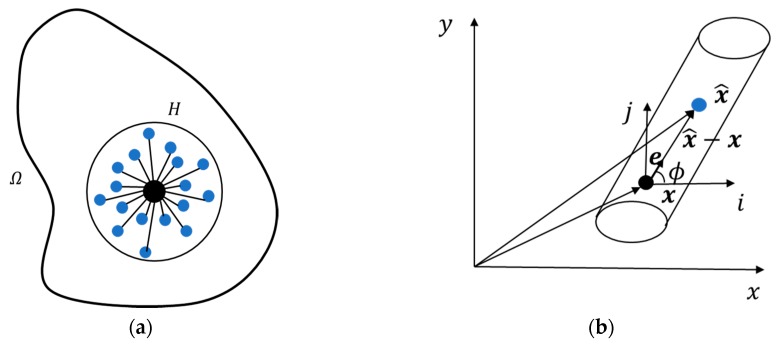
(**a**) The peridynamic horizon of a material point; (**b**) a peridynamic thermal bond between x and $\hat{x}$ in 2D.

As illustrated in Figure 1a, considering a body that occupies a region Ω consisting of material points, thermal transfer exists between each material point x and the associated neighboring points $\hat{x}$ within a finite domain, which is named horizon *H*. Each material point has the associated mass and volume. The thermal conductor between two interacting material points x and $\hat{x}$ is called ‘thermal bond’ or in short: t-bond (see Figure 1b). The peridynamic heat flux per unit volume transferring along the t-bond is assumed to depend on the relative location and the temperature difference between material points x and $\hat{x}$. The transient heat transfer equation in the form of a bond can be written as [26]:

(1) $$\rho c_{v}\dot{\Theta }\left(x,t\right)=\int_{H}^{}f_{h}\left(\hat{x},x,t\right)dV_{\hat{x}}+h_{s}\left(x,t\right)$$

where ρ and $c_{v}$ are the density and specific heat capacity of the material point x, respectively, and $h_{s}$ represents the heat generated per unit volume of the internal heat source. The kernel function (the function under the integral sign) $f_{h}\left(\hat{x},x,t\right)$ represents the heat exchange per unit volume of the material point $\hat{x}$ to the material point x, usually expressed in the following form [27]:

(2) $$f_{h}\left(\hat{x},x,t\right)=\{\begin{array}{c} \kappa \left(x,\hat{x}\right)\frac{\Theta \left(\hat{x},t\right)-\Theta \left(x,t\right)}{{\left\|\hat{x}-x\right\|}^{n}}\\ 0,\;\;\;\;\;\left\|\hat{x}-x\right\|>\delta \end{array},\;\left\|\hat{x}-x\right\|\leq \delta$$

where $\kappa \left(x,\hat{x}\right)$ represents the micro-conductivity of the t-bond between material points $\hat{x}$ and x. $\Theta \left(\hat{x},t\right)-\Theta \left(x,t\right)$ is the temperature difference between the two material points $\hat{x}$ and x. Here n is an integer, normally selected to be 0, 1, or 2 [27]. δ is the horizon size.

As described by Bobaru and Duangpanya [24], the PD heat flux vector between the material points x and $\hat{x}$ can be written as:

(3) $$q\left(x,t\right)=K\left(x,\hat{x}\right)\frac{\Theta \left(\hat{x},t\right)-\Theta \left(x,t\right)}{\left\|\hat{x}-x\right\|}e$$

where $K\left(x,\hat{x}\right)$ is the heat conductivity of the t-bond between material points x and $\hat{x}$, and where e=icosϕ+jsinϕ is the unit vector along the corresponding t-bond (see Figure 1b).

In the 2-D case, the classical heat flux vector between x and $\hat{x}$ along the t-bond because of their temperature difference can be written as [24]:

(4) $$q^{classical}\left(x,t\right)=K_{x}\left(x,\hat{x}\right)\frac{\Theta \left(\hat{x},t\right)-\Theta \left(x,t\right)}{\left\|\hat{x}-x\right\|}cos\phi i+K_{y}\left(x,\hat{x}\right)\frac{\Theta \left(\hat{x},t\right)-\Theta \left(x,t\right)}{\left\|\hat{x}-x\right\|}sin\phi j$$

where $K_{x}\left(x,\hat{x}\right)$ and $K_{y}\left(x,\hat{x}\right)$ are the thermal conductivities of the t-bond ($x,\hat{x}$) respectively in *x* and *y* directions.

In the bond-based peridynamics transient heat transfer theory, assuming that the thickness of the plate is h, the PD heat flux between x and $\hat{x}$ along the t-bond can be obtained from [24]:

(5) $$q^{Peri}\left(x,t\right)=\int_{H^{+}}^{}\left\|\hat{x}-x\right\|f_{h}\left(\hat{x},x,t\right)dV_{\hat{x}}=\int_{H^{+}}^{}\kappa \left(x,\hat{x}\right)\frac{\Theta \left(\hat{x},t\right)-\Theta \left(x,t\right)}{{\left\|\hat{x}-x\right\|}^{n-1}}dV_{\hat{x}}$$

The natural value for the parameter n in Equation (5) was 2 in the literature [24]. Here we let n be 0, 1 and 2 to get the different types of kernels mentioned before in the 2-D case.

Two kinds of micro-conductivity functions are considered. The first one is a constant:

(6) $$\kappa \left(x,\hat{x}\right)=\kappa_{0}\left(x,\hat{x}\right)$$

Another one varies linearly over the horizon size called conical micro-conductivity function:

(7) $$\kappa \left(x,\hat{x}\right)=\kappa_{1}\left(x,\hat{x}\right)\left(1-\frac{\left\|\hat{x}-x\right\|}{\delta }\right)$$

**Figure 2:**
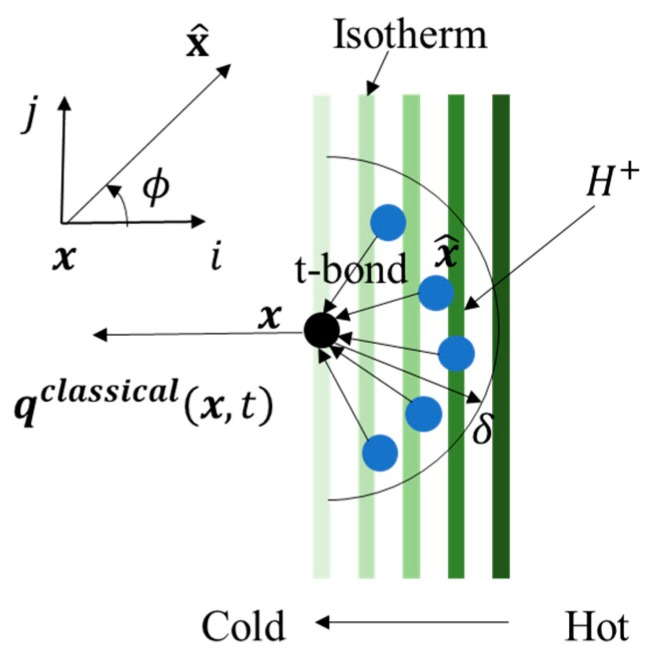
A liner varying temperature field around point x.

The micro-conductivity is obtained by equating the peridynamic heat flux with the classical heat flux. The temperature field is assumed to be linearly distribution of the following form Θ(x,y,t)=ax+b, where a and b are constants, as shown in Figure 2. The classical heat flux and peridynamic heat flux perpendicular to the isothermal line at the point *x* are respectively expressed as:

(8) $$q^{classical}\left(x,t\right)=K\left(x,\hat{x}\right)\frac{\partial \Theta \left(x,y\right)}{\partial x}=K\left(x,\hat{x}\right)a$$

.

With the constant shape factor, substituting Equation (6) into Equation (5), the PD heat flux can be written as:

(9) $$q^{Peri}\left(x,t\right)=\frac{\delta^{4-n}}{2\left(4-n\right)}\kappa_{0}\left(x,\hat{x}\right)ah\pi$$

With the ‘‘triangular’’ shape factor, substituting Equation (7) into Equation (5), the PD heat flux can be written as:

(10) $$q^{Peri}\left(x,t\right)=\frac{\delta^{4-n}}{2\left(4-n\right)\left(5-n\right)}\kappa_{1}\left(x,\hat{x}\right)ah\pi$$

Equating (9), (10) with the classical flux in (8), gives:

(11) $$\kappa_{0}\left(x,\hat{x}\right)=\frac{2\left(4-n\right)}{\pi h\delta^{4-n}}K\left(x,\hat{x}\right)$$

(12) $$\kappa_{1}\left(x,\hat{x}\right)=\frac{2\left(4-n\right)\left(5-n\right)}{\pi h\delta^{4-n}}K\left(x,\hat{x}\right)$$

For the conical micro-conductivity, $\kappa \left(x,\hat{x}\right)$ decreases linearly with the bond length, making the PD model closer to the classical localized model, reducing the integral error caused by the PD surface effect. In this paper, in order to make the integral error more obvious, the constant micro-conductivity and the integer n=1 is adopted. In the next section, a peridynamic model for transient heat conduction in the FGM with cracks is given.

## 3. A Peridynamic Transient Heat Conduction Model for FGMs

### 3.1. Numerical Discretization of FGMs in 2D

Functionally graded materials are basically heterogeneous materials, which are considered as composites composed of two-phase materials, the volume fraction of which gradually changes from one end to the other. For the convenience of analysis, we assume that the material parameters such as density, specific heat capacity, and heat transfer coefficient of FGMs are functions of spatial coordinates, and the local material parameters can be regarded as fixed constants. They can be expressed as:

(13) $$\rho \left(x,y\right)=P\left(\rho_{0},x,y\right)$$

(14) $$c\left(x,y\right)=P\left(c_{0},x,y\right)$$

(15) $$K\left(x,y\right)=P\left(K_{0},x,y\right)$$

where *P* represents the gradient function of the material parameter. Cheng et al. proposed a peridynamic model of FGMs, which calculated the peridynamic micro-modulus of the bond by the mean values of the material properties at the two interacting material points [30]. The equivalent micro-conductivity $\kappa_{eff}$ of the t-bond between the two material points x and $\hat{x}$ can be approximated as:

(16) $$\kappa_{eff}\left(x,\hat{x}\right)=\frac{6}{\pi h\delta^{3}}K_{eff}\left(x,\hat{x}\right)$$

where $K_{eff}\left(x,\hat{x}\right)=0.5\left(K\left(x,y\right)+K\left(\hat{x},\hat{y}\right)\right)$. The bond-based PD transient heat conduction equation of FGMs can be discretely expressed as:

(17) $$\rho \left(x_{i}\right)c_{v}\left(x_{i}\right)\dot{\Theta }\left(x_{i},t\right)=\overset{n}{\underset{j=1}{\sum }}\kappa_{eff}\left(x_{i},x_{j}\right)\frac{\Theta \left(x_{j},t\right)-\Theta \left(x_{i},t\right)}{\left\|x_{j}-x_{i}\right\|}V_{x_{j}}+h_{s}\left(x_{i},t\right)$$

where i represents the point of interest, j represents the family members of point i and n is the total number of all points j.

### 3.2. Dirichlet Boundary Condition

**Figure 3:**
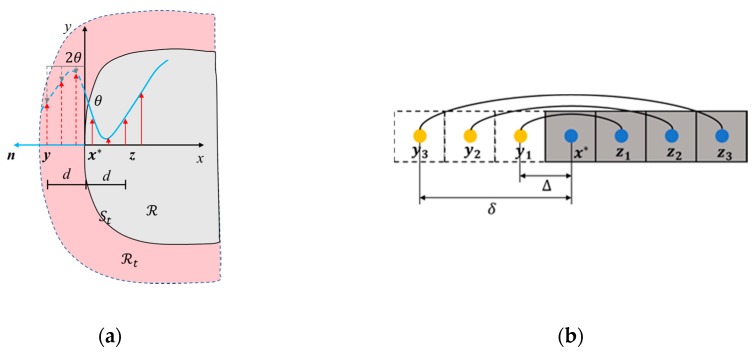
(**a**) Material point x and its image in fictitious region; (**b**) the temperatures of the fictitious nodes be distributed.

In general, the PD method causes the boundary effect by applying a fixed temperature directly to the material points at the boundary [23,24]. This boundary condition application method will result in incomplete neighborhood of the material points near the boundary, thereby weakening the boundary condition. This paper refers to the method of the fictitious nodes [26] when dealing with the Dirichlet boundary conditions. Based on his research, the extent of the virtual boundary layer to be equal to the horizon, *δ* in order to ensure that the prescribed temperatures sufficiently reflected in the actual material region. As shown in Figure 3, Dirichlet boundary condition, $\Theta \left(x^{*},t\right)$ can be applied in the virtual boundary layer, ${ℛ}_{t}$ along the boundary of the body surface, as:

(18) $$\Theta \left(y_{i},t+∆t\right)=2\Theta \left(x^{*},t+∆t\right)-\Theta \left(z_{i},t\right),x^{*}\in S_{t},\;y\in {ℛ}_{t},z\in ℛ,\;i=1,2,3$$

where $z_{i}$ represents the location of a material point in ℛ, and $x^{*}$ is the position of a point on the body surface, $S_{t}$. $\Theta \left(z_{i},t\right)$ represents the temperature of the material point in the body, which is obtained by solving the PD transient heat conduction equation. $\Theta \left(y_{i},t\right)$ represents the temperature of the material point in the virtual layer. When time equals t+∆t, $\Theta \left(x^{*},t+∆t\right)$ is known from the analytical boundary condition, so we can get the $\Theta \left(y_{i},t+∆t\right)$ by the Equation (1). During numerical simulation, $\Theta \left(y_{i},t\right)$ can be calculated at each time step in the virtual boundary layer.

### 3.3. Modeling of Insulated Crack

**Figure 4:**
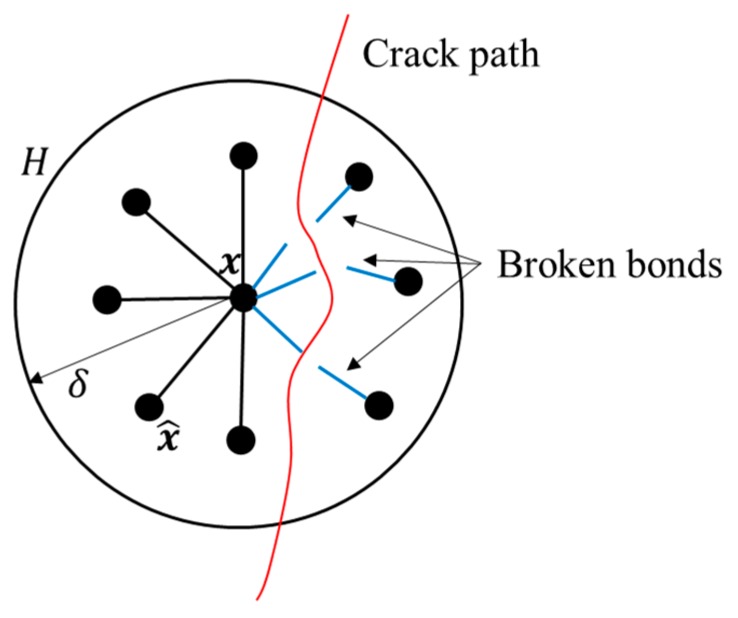
Crack over the horizon of material point x.

To simulate transient heat conduction for FGMs with cracks, cracks need to be modelled by PD. As shown in Figure 4, the partial t-bonds in the horizon of material point x were broken. Referring to Bobaru and Duangpanya [24], the kernel function $f_{n}\left(\hat{x},x,t\right)$ in Equation (2) is set as a “history-dependent” function:

(19) $${\tilde{f}}_{n}\left(\hat{x},x,t\right)=f_{n}\left(\hat{x},x,t\right)\mu \left(t,\left\|\hat{x}-x\right\|\right)$$

where μ is a history-dependent scalar valued function, which is defined as:

(20) $$\mu \left(t,\left\|\hat{x}-x\right\|\right)=\{\begin{array}{c} 1\;\;\;\;\;\;\;\;\;\;\;\;\;\;\;\;if\;bond\;is\;intact\;\\ \alpha \left(t,\xi ,\eta \right)\;\;\;\;\;\;otherwise\;\;\;\;\;\;\;\;\;\;\;\;\;\;\;\;\; \end{array}$$

.

In this paper, it is assumed that the crack is an insulated crack, and the heat conduction of the t-bonds crossing the crack were completely suppressed. The scalar valued function α is zero if the t-bond crosses the insulated crack.

### 3.4. Surface Correction

The micro-conductivity parameters are determined by equating the peridynamic heat flux values with the classical heat flux values. The micro-conductivity parameters are calculated by assuming integration over a full horizon. Near a domain boundary, the peridynamic horizon is not complete. Therefore, the value of κ needs to be corrected if the material points near the free surfaces or material interface.

A number of methods/algorithms have been proposed recently for surface correction. Le and Bobaru [30] discuss in detail the accuracy and computational efficiency of multiple surface correction methods for elasticity and fracture. These methods include the volume method [31], the force density method [32], the energy method [32], the force normalization [33], the position-aware method [34], and fictitious nodes method [26,32]. For transient heat conduction problem, we reference the surface correction factor g(x) proposed in [26] to reduce the PD surface effect based on the energy method. It is obtained by comparing the PD thermal potential with the corresponding classical thermal potential energy for simple temperature distribution.

The classical thermal potential formulation at point x can be written as [26]:

(21) $$Z_{\infty }\left(x\right)=\frac{1}{2}G\cdot K\left(x\right)G$$

where K(x) is the thermal conductivity and G=∇Θ.

The PD thermal potential is the integral over all microthermal potentials associated with this point, and is expressed as [26]:

(22) $$Z\left(x\right)=\frac{1}{2}\int_{H}^{}\kappa_{eff}\left(x,\hat{x}\right)\frac{{\left(\Theta \left(\hat{x},t\right)-\Theta \left(x,t\right)\right)}^{2}}{2\left\|\hat{x}-x\right\|}dV_{\hat{x}}$$

where $\kappa_{eff}$ is the equivalent micro-conductivity of the t-bond between the two material points x and $\hat{x}$. The correction factor of the material points in the body can be determined by [26]:

(23) $$g\left(x\right)=\frac{Z_{\infty }\left(x\right)}{Z\left(x\right)}$$

Therefore, the discretized thermal diffusion Equation (17) containing the surface correction factor for point x can be written:

(24) $$\rho \left(x_{i}\right)c_{v}\left(x_{i}\right)\dot{\Theta }\left(x_{i},t\right)=\overset{n}{\underset{j=1}{\sum }}g\left(x_{i},x_{j}\right)\kappa_{eff}\left(x_{i},x_{j}\right)\frac{\Theta \left(x_{j},t\right)-\Theta \left(x_{i},t\right)}{\left\|x_{j}-x_{i}\right\|}V_{x_{j}}+h_{s}\left(x_{i},t\right)$$

where $g\left(x,\hat{x}\right)=\left(g\left(x\right)+g\left(\hat{x}\right)\right)/2$.

For the material points close to insulated cracks, the peridynamic horizon is incomplete due to the partial t-bond disconnection (see Figure 4). In principle, in order to match the conductivity parameter of a material point with complete horizon while integrating over a smaller area (due to the presence of an insulating crack), one has to increase the micro-conductivity value of the t-bond to compensate for the reduction of integration area.

Considering the partial t-bond of the material point x in the horizon is broken, the thermal potential energy expressed by the Equation (22) can be written as:

(25) $$Z\left(x\right)=\frac{1}{2}\int_{H}^{}\mu \left(t,\left\|\hat{x}-x\right\|\right)\kappa_{eff}\left(x,\hat{x}\right)\frac{{\left(\Theta \left(\hat{x},t\right)-\Theta \left(x,t\right)\right)}^{2}}{2\left\|\hat{x}-x\right\|}dV_{\hat{x}}$$

The classical thermal potential is shown in Equation (21), so the surface correction factor can also be expressed by Equation (23). When dealing with transient heat conduction problems with dynamic cracks, new t-bonds will break near the crack as the crack grows. The PD thermal potential energy expressed by Equation (25) will change. The correction factor needs to be calculated at each step according to Equation (23). However, for transient thermal conduction problems with static cracks or without cracks, this parameter only needs to be calculated in the first time step and remains constant after the first step.

## 4. Numerical Convergence Studies

We analyse the convergence of the proposed peridynamic model for FGMs by the following example of a 2-D FGM plate without any crack. The convergence tests in the limit of nonlocal parameter going to zero are performed by comparing the peridynamic solutions with the classical analytic solution.

### 4.1. The Peridynamic Model for FGMs

In the literature [19], the bond-based PD model for dynamic fractures in FGMs was verified by elastic wave propagation and comparisons with analytical results for the classical model. In order to verify the reliability of the PD heat conduction model of FGMs proposed in Section 3, the temperature field and heat flux of the following example are calculated and compared with the analytical solution.

**Figure 5:**
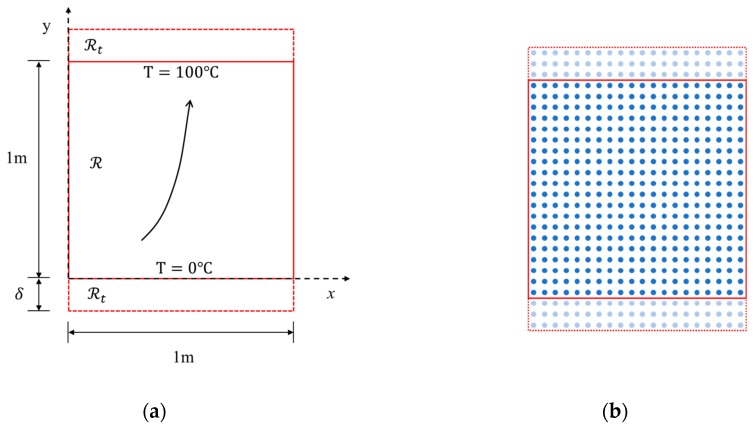
Example 1: (**a**) The geometry and the boundary conditions of the FGM plate; (**b**) discretization scheme for PD material points of the FGM plate.

As shown in Figure 5a, the FGM plate has a length and width of 1.0 m, a thickness of 0.01 m, and an initial temperature of 0 °C. The top edge is fixed at a constant temperature of 100 °C. The bottom edge is 0 °C. The left and right edges are well insulated (zero normal flux). The geometry details and the boundary conditions are shown in Figure 5a. Figure 5b is the PD discretization model of the FGM plate.

The thermal conductivity and the specific heat of FGMs are defined by

(26) $$K\left(x,y\right)=5.0e^{\lambda y}\;W/\left(m\cdot K\right)$$

(27) $$c\left(x,y\right)=1.0e^{\lambda y}\;J/\left(kg\cdot K\right)$$

where exponential parameters λ=3 m^−1^, and the density $\rho =1.0\;\mathrm{kg}/m^{3}$.

The analytical solution for temperature filed is given in [2] by

(28) $$\begin{array}{c} \Theta \left(x,y,t\right)=T\frac{1-e^{-2\beta y}}{1-e^{-2\beta L}}+\overset{\infty }{\underset{n=1}{\sum }}B_{n}sin\frac{n\pi y}{L}e^{-\beta y}e^{-\left(n^{2}\pi^{2}/L^{2}+\beta^{2}\right)\alpha t}\\ B_{n}=\frac{2Te^{\beta L}n\pi cos\left(n\pi \right)}{\beta^{2}L^{2}+n^{2}\pi^{2}} \end{array}$$

where L is the length of the plate in the y-direction, β=λ/2=1.5 m^−1^, α=5.0. The distribution of heat flux can be obtained by the derivative of temperature with respect to time

(29) $$\begin{array}{c} q\left(x,y,t\right)=2K\left(x,y\right)\beta T\frac{e^{-2\beta y}}{1-e^{-2\beta L}}\\ \;\;\;\;\;\;\;+\overset{\infty }{\underset{n=1}{\sum }}K\left(x,y\right)B_{n}\left(\frac{n\pi }{L}cos\frac{n\pi y}{L}-\beta sin\frac{n\pi y}{L}\right)e^{-\beta y}e^{-\left(n^{2}\pi^{2}/L^{2}+\beta^{2}\right)\alpha t} \end{array}$$

**Figure 6:**
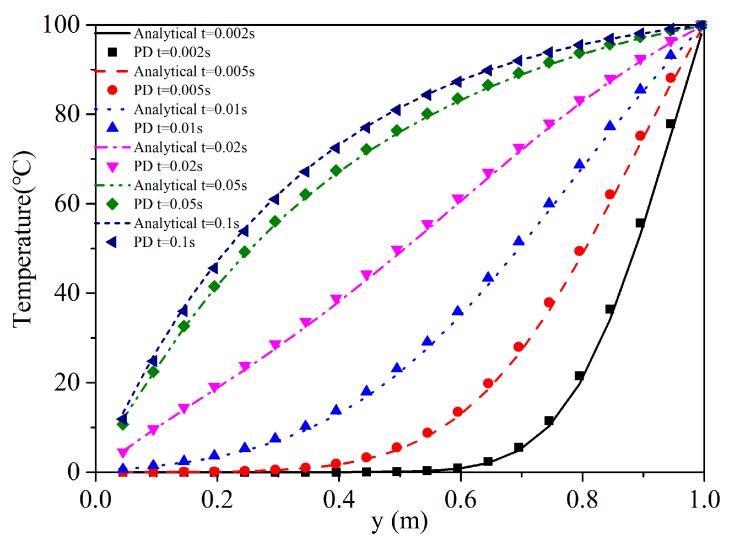
Temperature along the line *x* = 0.495 m on various time levels of different methods.

**Figure 7:**
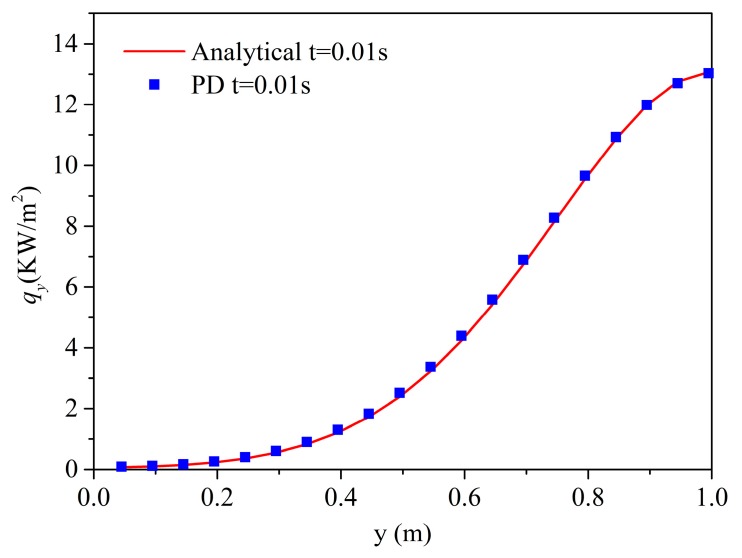
Heat flux along the line *x* = 0.495 m at t = 0.01 s of different methods for the example.

The *x* and *y* directions of the model are discretized into 100 material points, the point spacing ∆=0.01 m, the horizon size δ=3∆, and the time step $∆t=10^{-5}$ s. In Figure 6, the temperature distribution along the line *x* =0.495 m is compared with the solution obtained by analytical method. The solution is plotted at different times: 0.002, 0.005, 0.01, 0.02, 0.05, and 0.1 s, to observe its transient heat transfer characteristics. The vertical heat flux comparison at *t* = 0.05 s along the line *x* =0.495 m is plotted in Figure 7. The numerical results agree very well with the analytical solution.

### 4.2. Convergence Study

In order to investigate the effect of surface correction on the numerical accuracy of peridynamic heat conduction model for FGMs, the relative difference of our nonlocal solutions with the classical solution introduced in [24] can be defined as:

(30) $$\frac{\left\|\theta_{classical}-\theta_{peri}\right\|}{\left\|\theta_{classical}\right\|}$$

**Figure 8:**
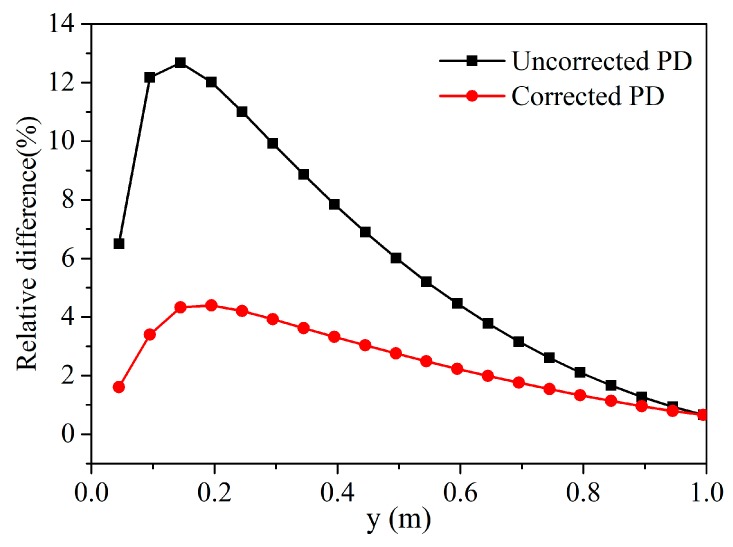
Comparison of the relative difference in temperature along the line *x* = 0.495 m at *t* = 0.01 s.

We compared the relative differences in temperature between the corrected peridynamic model and the uncorrected peridynamic model. As we can see from Figure 8, the relative difference after correction is obviously smaller than uncorrected PD. Therefore, it can be said that the correction of the surface effect improves calculation accuracy.

In order to observe the convergence behavior of the PD transient heat conduction model after surface correction to the classical analytical solution of the FGM plate, the m convergence and *δ* convergence (see [35]) of this model are studied respectively. The temperature of the material point with the coordinates of (0.495 m, 0.495 m) in the middle of the FGM plate at *t* = 0.01 s is used to evaluate convergence of the PD solutions to the classical solution.

**Figure 9:**
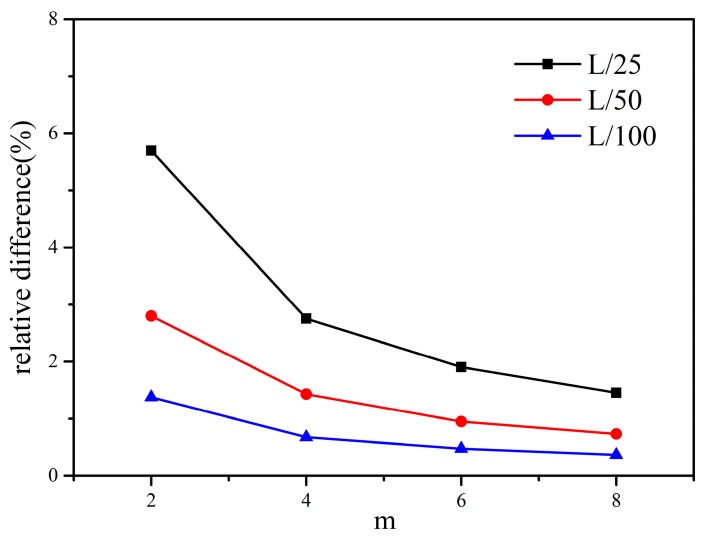
The *m*-convergence for three different horizon sizes (*L* divided by 25, 50, and 100).

**Figure 10:**
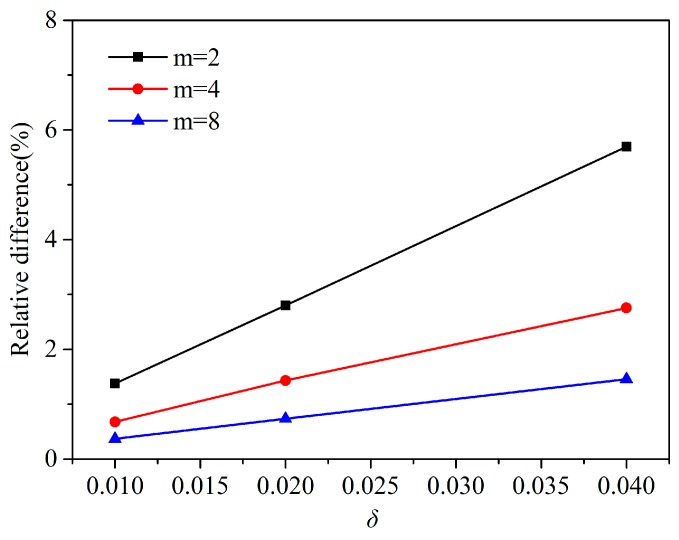
The δ-convergence for three different nonlocal ratio (*m* = 2, 4, 8).

**Figure 11:**
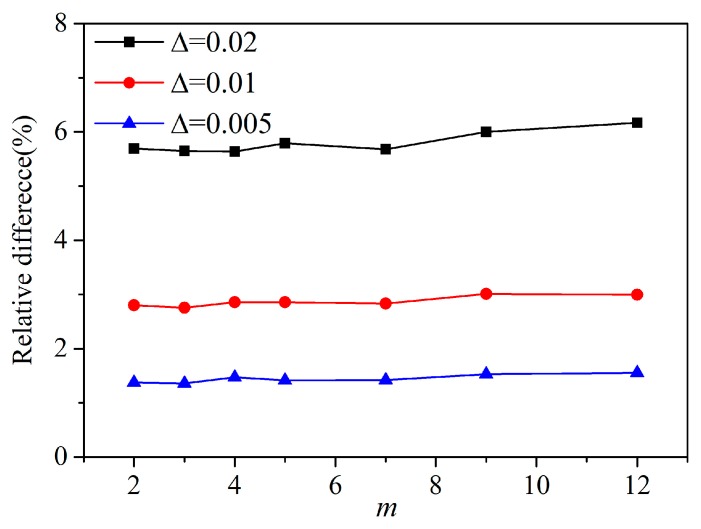
The *m*-convergence for three different point spacing (∆ = 0.02, 0.01, and 0.005 m).

In terms of *m*-convergence, as shown in Figure 9, the fixed horizon δ is equal to 0.04, 0.02, and 0.01 m, the results show that when δ is constant, the relative difference tends to decrease with increasing the nonlocal ratio, m=δ/∆. In terms of *δ*-convergence, the nonlocal ratio *m* is equal to 2, 4, and 8, it can be seen that the relative difference tends to decrease with decreasing horizon size *δ* in Figure 10. When *δ* = 0.01 m and *m* = 4, 8 and *δ* = 0.02 m and *m* = 8, the numerical calculation has higher precision, and the relative difference is less than 1%. Another type of *m*-convergence is also used for analysis, the point spacing ∆ is fixed and the nonlocal ratio m is changed. As shown in Figure 11, we analyse the *m*-convergence for three different point spacing: ∆ = 0.02, 0.01, and 0.005 m. The relative difference of the numerical calculation changes very little with increasing *m*, while the point spacing is fixed. The relative difference when *m* = 3 is slightly less than when m is equal to some other values. The relative difference decreases as the material point spacing decreases.

In PD analysis, the computational time increases with the point spacing decreasing and the nonlocal ratio increasing. Thus, it is important to use the optimal point spacing for the desired computational efficiency and accuracy. Based on the convergence study, the family of each point is established by the horizon size of 3 associated with the grid spacing of 0.01 m.

### 4.3. Discretization Schemes for PD Material Points

**Figure 12:**
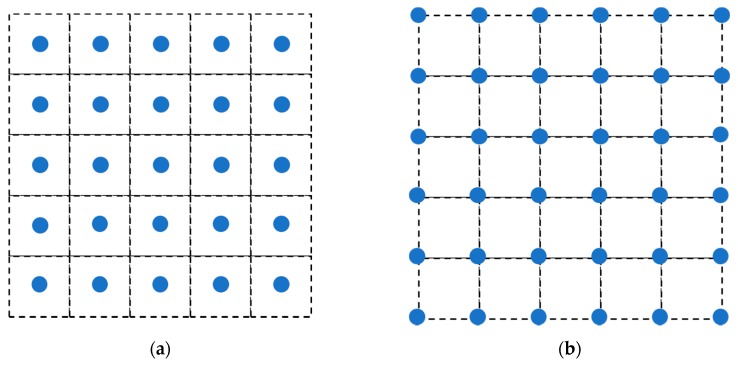
Discretization schemes for the PD material points of the FGM plate: (**a**) scheme Ⅰ: points not on the boundary. (**b**) scheme Ⅱ: points on the boundary.

The domain is divided into square grids, with each material point at the center of the grid. There are two ways to assign the material points positions. One of these is shown in Figure 5b, the center of the material points closest to the boundary is not on the boundary of the FGM plate, but inside the body. As shown in Figure 12a, it is called discretization scheme Ⅰ. When Dirichlet boundary condition is applied to the boundary of the FGMs plate, the temperature is actually applied to the material point with a certain volume and it has an error with the true Dirichlet boundary condition. Based on the above reason, we use discretization scheme Ⅱ to keep the center of the material point on the sample boundary, as shown in Figure 12b. The other parameters are the same as in Section 4.1. and the calculated result is compared with the scheme Ⅰ.

**Figure 13:**
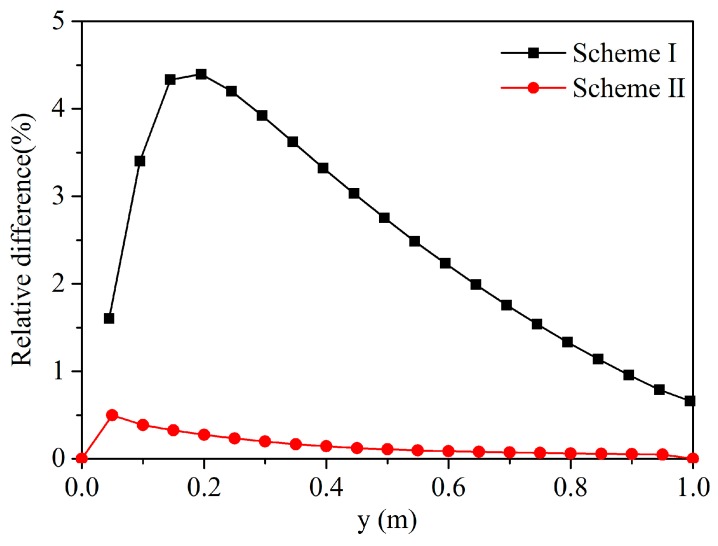
Comparison of the relative difference in temperature, between the solution of scheme Ⅱ along the line *x* = 0.5 and the solution of scheme Ⅰ along the line *x* = 0.495 m at *t* = 0.01 s.

As we can see from Figure 13, the PD scheme Ⅱ has a lower relative difference than the scheme Ⅰ, and the relative error of each position point is smaller. Table 1 is the comparison of the temperature between the discretization scheme Ⅱ and the analytical solution at *t* = 0.01 s, *t* = 0.02 s along *x* = 0.5 m line, and the relative differences of the scheme Ⅱ are all below 0.5%. Therefore, the numerical solution obtained by the scheme Ⅱ which align the material points on the sample’s boundary is closer to the analytical solution.

## 5. Transient Heat Conduction of the FGM Plate with Cracks

### 5.1. Transient Heat Conduction of the FGM Plate with Static Crack

**Figure 14:**
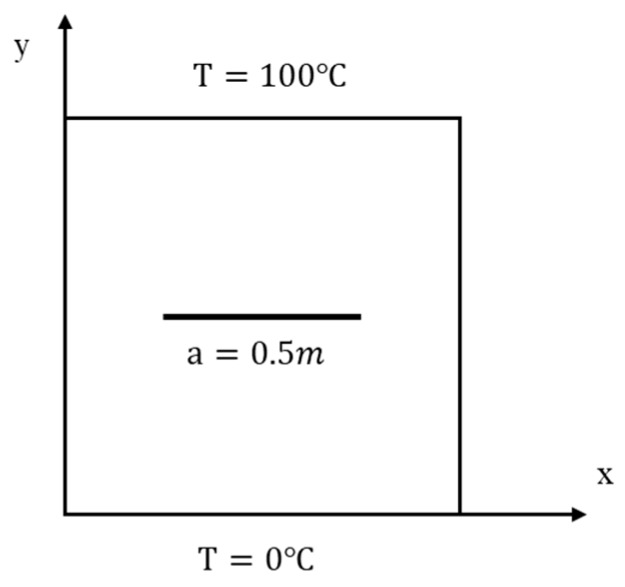
Example 2: geometry and boundary conditions of the FGM plate with a horizontal static crack.

As shown in Figure 14, the FGM plate has a horizontal static insulated crack with a length of 0.5 m. Material properties, the geometry details and the boundary conditions are the same as the previous example.

The example is discretized by the scheme Ⅱ and the model (PD2) considering the micro-conductivity correction of the material points close to the crack is adopted. Results from the PD1 (the PD model without considering the micro-conductivity correction of material points close to the crack) and the classical finite element method (FEM) are used for comparison.

**Figure 15:**
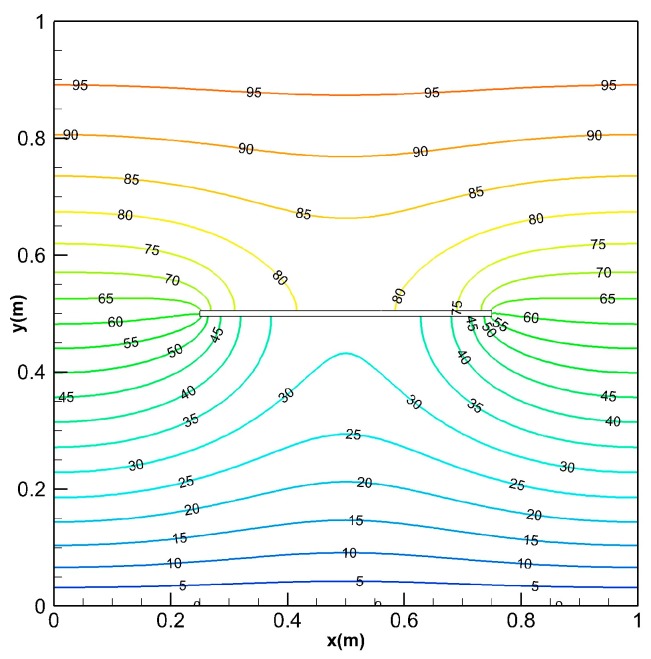
The temperature field at *t* = 0.03 s for the classical model using FEM discretization with 11,068 linear quadrilateral elements.

**Figure 16:**
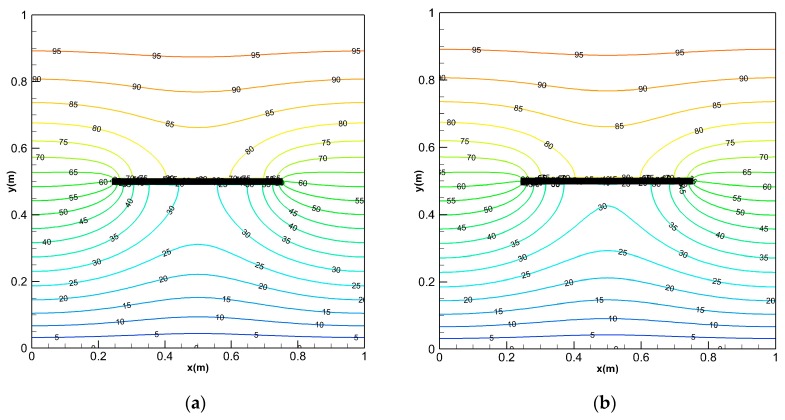
The temperature field at *t* = 0.03 s obtained with (**a**) PD1; (**b**) PD2.

**Figure 17:**
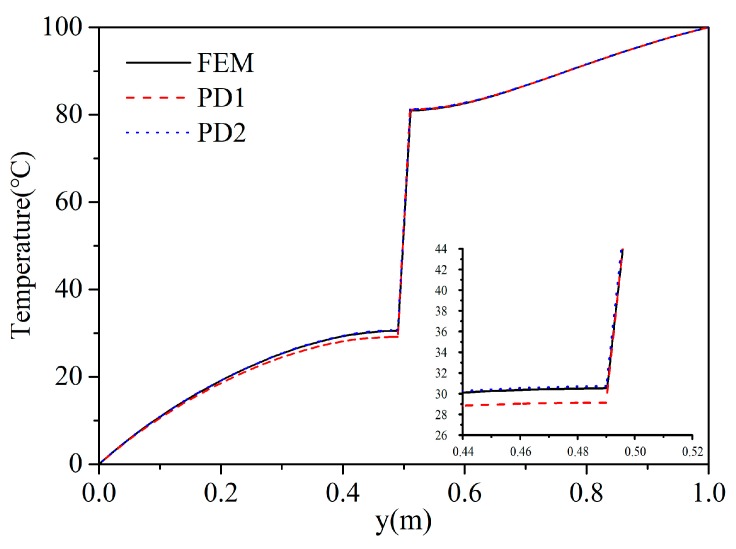
Temperature comparison along the line *x* = 0.5 m at *t* = 0.03 s from FEM, PD1 and PD2.

Figure 15 shows the temperature field of FEM result at *t* = 0.03 s. Figure 16 shows the temperature distribution of PD1 and PD2 at *t* = 0.03 s. At this time, the temperature in the FGM plate has not reached a steady state. It can be observed from Figure 15 and Figure 16 that the temperature contours of PD2 and FEM are almost identical. But, the temperature of PD1 at the bottom of the crack is lower than the temperature obtained by FEM. This phenomenon can also be observed by comparing the temperature results of the FGM plate along the line *x* = 0.5 m at *t* = 0.03 s (see Figure 17). The reason for the above results can be analyzed as follows. The micro-conductivity of the material points close to crack from the PD1 method is lower than the true value, which leads to a decrease in the heat transfer efficiency. Hence, our proposed PD model which considers the micro-conductivity correction of the material points close to the crack can simulate transient heat conduction of FGMs plate with insulated crack exactly.

### 5.2. Transient Heat Conduction of the FGM Plate with Dynamic Horizontal Crack

In the practical coupled thermo-mechanical engineering problems, the initiation and propagation of cracks is often abrupt and uncontrollable. The classical numerical method is difficult to deal with this sudden spatial discontinuity problem because spatial derivatives don’t exist over discontinuities. Peridynamics fundamentally eliminates these difficulties since spatial derivatives do not appear in the formulation.

**Figure 18:**
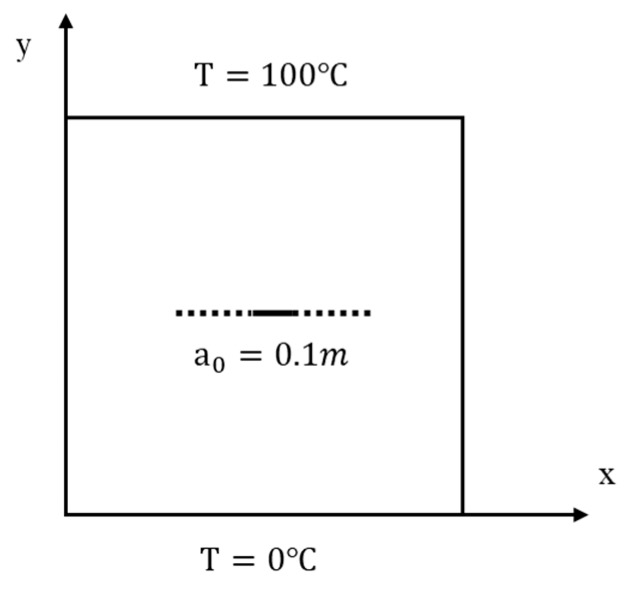
Example 3: Geometry and boundary conditions of the FGM plate with a horizontal dynamic crack.

To demonstrate the advantage of PD, we consider the problem of transient heat conduction of the FGM plate with a horizontal dynamic crack. The growth of the crack is imposed by us here. As shown in Figure 18, consider the pre-existing adiabatic horizontal crack in the middle of the FGM plate. The initial length of the crack is 0.1 m. The crack can be dynamically extended along the *x* direction. The rate of 80 m/s is extended to both sides, and the final crack length is 0.5 m at *t* = 0.05 s. The material parameters of the FGM plate are the same as example 1. The geometry details and the boundary conditions are the same as the first example. It is assumed that the extended crack can cut the heat conduction of the t-bond passing through the crack and we consider the micro-conductivity correction of the material points close to crack introduced in Section 3.4.

**Figure 19:**
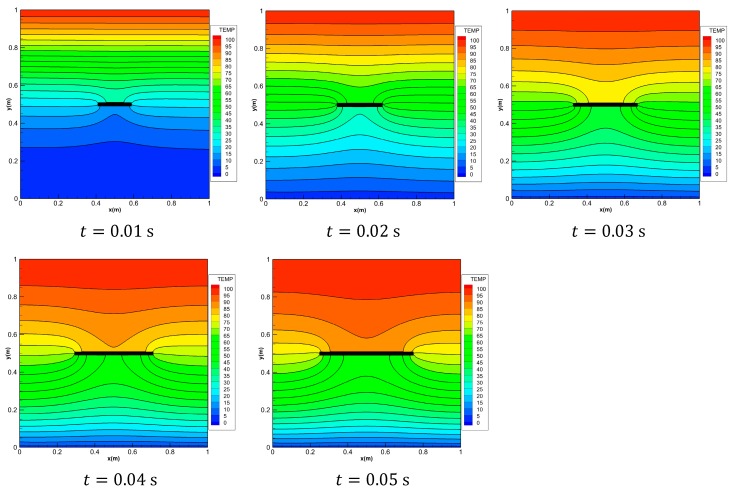
Temperature distributions on FGMs plate with growing crack.

The solutions are shown in Figure 19, the temperature distributions of the FGM plate with growing crack are shown at 0.01 s, 0.02 s, 0.03 s, 0.04 s and 0.05 s, respectively. With the increase of time, the dynamic crack become longer and hinders the heat conduction of the t-bond passing through the crack. As observed in these Figures, it is obvious that the presence of the crack causes discontinuous temperature fields above and below the crack surface. The temperature values below the crack drop suddenly because the insulated cracks behave as a thermal barrier in the FGM plate. It is also observed that the temperature gradient near the end of the crack is higher than its neighborhood. According to the thermodynamics knowledge, the position with larger temperature gradient always generates larger thermal stress, then leads to crack growth. It is worth noting that the shielding effect induced by the dynamic horizontal crack is correctly captured by the peridynamic simulation.

### 5.3. Transient Heat Conduction of the FGM Plate with Dynamic Intersecting Cracks

**Figure 20:**
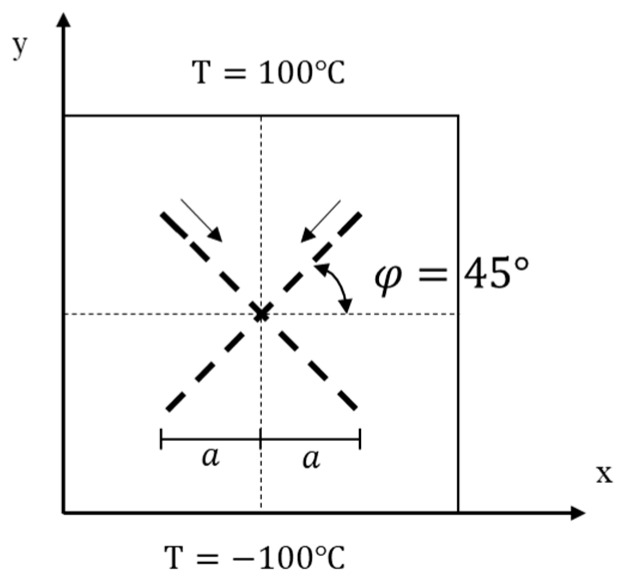
Example 4: Geometry and boundary conditions of the FGM plate with dynamic intersecting crack.

Under thermal shock, FGMs will generate complex cracks, including some interacting cracks. As shown in Figure 20, we consider the problem of transient heat conduction for the FGM plate in which two insulating cracks initiation, propagation and intersect. The geometry of the plate is the same as in the first example. The growth of two cracks is imposed by us. The two cracks begin to grow at time *t* = 0 s with a constant extension velocity 0.2828 m/s. A symmetric x-like crack is obtained at the end of the simulation with the spacing a=0.25 m. The initial temperature of the FGM plate is 0 °C. The left and right boundaries are insulated and temperatures of ±100 °C are imposed on the top and bottom boundaries, respectively (see Figure 20). To investigate the influence of the material gradation on the temperature variation, three different exponential parameters λ= 0, 1 and 3 $m^{-1}$ are chosen for comparison in numerical calculation of the FGMs.

**Figure 21:**
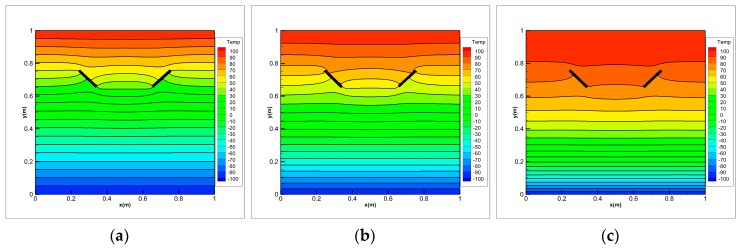
Temperature distribution on FGMs plate with dynamic intersecting cracks for (**a**) λ=0, (**b**) λ=1 and (**c**) $\lambda =3\;m^{-1}$ at *t* = 0.5 s.

**Figure 22:**
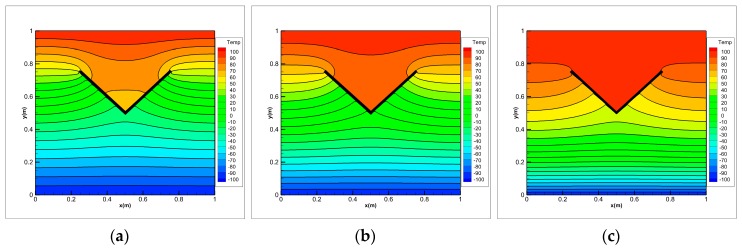
Temperature distribution on FGMs plate with dynamic intersecting cracks for (**a**) λ=0, (**b**) λ=1 and (**c**) $\lambda =3\;m^{-1}$ at *t* = 1.25 s.

**Figure 23:**
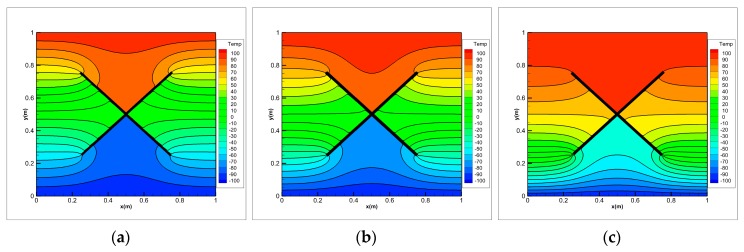
Temperature distribution on FGMs plate with dynamic intersecting cracks for (**a**) λ=0, (**b**) λ=1 and (**c**) $\lambda =3\;m^{-1}$ at *t* = 2.5 s.

**Figure 24:**
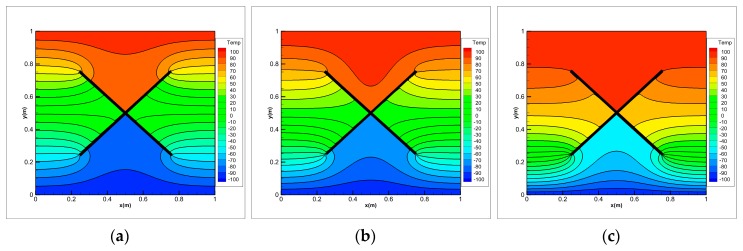
Temperature distribution on FGMs plate with dynamic intersecting cracks for (**a**) λ=0 , (**b**) λ=1 and (**c**) $\gamma =3\;m^{-1}$ at *t* = 5 s.

The temperature distributions at different times for three values of the exponential parameter λ are shown in Figure 21, Figure 22, Figure 23 and Figure 24. The steady-state results are given at *t* = 5 s. As the cracks grow and the crack gap closes, the temperature rises above the V-shaped crack and drops below it. Once the cracks intersect with each other and continue to grow, the temperature values jump around the intersection point of x-like crack in FGM plate. Before the steady state is reached, the temperature difference between the top and bottom of the intersection point increases with the calculation time. When λ= 0, the material becomes homogeneous and the temperature is symmetrically up and down when *t* = 5 s.

Comparing the results of three different exponential parameters, it is found that the temperature increases along with an increase in λ-values (or equivalently in thermal conductivity) at the same moment. It can also be found that as the λ-values increase, the temperature gradient at the crack tip of the FGM plate decreases before the cracks intersect, but it increases after the cracks intersect. From these pictures, difference between the homogeneous material and the FGMs can be observed. The shielding effect induced by the dynamic intersecting cracks is correctly captured by the peridynamic solution.

## 6. Conclusions and Future Work

In this paper, a peridynamics model for transient heat conduction in functionally graded materials with insulated cracks was introduced. To eliminate the peridynamic surface effect near a domain boundary and insulated cracks, the improved surface correction factor containing the crack was presented by referring to the original surface correction factor.

Subsequently, the present model was verified by comparison with analytical solutions for FGMs without cracks. We compared the relative differences of temperature between the corrected PD model and the uncorrected PD model, it was observed that the numerical calculation accuracy was improved by surface correction. The convergence of the model was studied. The influences of the horizon and the nonlocal ratio on the corrected PD model were investigated by measuring the relative difference. We showed that as the horizon decreases, the PD solutions converge to the analytical solution. However, the relative difference of the numerical calculation changed very little with an increasing nonlocal ratio m, while the point spacing is fixed. By comparing two different discretization schemes for PD material points, it was found that when the center of the boundary material points is located on the boundary, the PD solution is more accurate.

For the transient heat conduction of FGMs with cracks, we introduced a method of considering the surface correction of the material points close to an insulated crack to improve the accuracy of numerical calculation near the crack. We then solved the transient heat conduction of FGMs with horizontal static cracks. The results showed better agreement with results from FEM than the results from the uncorrected PD model. The transient heat conduction of the FGM plate with dynamic horizontal crack and dynamic intersecting cracks was simulated. The shielding effect of the thermal conduction of the temperature field caused by the dynamic crack propagation was correctly captured. The developed corrected PD heat conduction model can accurately simulate the transient heat conduction problem of functionally graded materials with cracks.

We plan to study coupling response between the thermal and mechanical of FGMs under thermal shock loading, and simulate the crack initiation, propagation and failure process of FGMs.

## Figures and Tables

**Table 1:** Comparison of the temperature of the discretization scheme Ⅱ with the analytical solution along the line x = 0.5 m at t = 0.01 s, t = 0.02 s.

*y* (m)	*t* = 0.01 s			*t* = 0.02 s		
	Analytical (°C)	PD (°C)	Relative Difference	Analytical (°C)	PD (°C)	Relative Difference
0.0	0.0000	0.0000	0.0000%	0.0000	0.0000	0.0000%
0.1	1.3779	1.3832	0.3857%	9.9293	9.9394	0.1015%
0.2	3.4151	3.4245	0.2764%	18.8305	18.8416	0.0589%
0.3	7.0249	7.0388	0.1974%	28.0408	28.0538	0.0464%
0.4	13.0901	13.1088	0.1427%	38.1238	38.1393	0.0406%
0.5	22.3446	22.3686	0.1077%	49.0987	49.1169	0.0370%
0.6	35.0706	35.1008	0.0861%	60.6112	60.6320	0.0344%
0.7	50.7914	50.8282	0.0724%	72.0826	72.1056	0.0319%
0.8	68.1695	68.2119	0.0622%	82.8518	82.8759	0.0291%
0.9	85.2493	85.2942	0.0527%	92.3084	92.3321	0.0258%
1.0	100.0000	100.0000	0.0000%	100.0000	100.0000	0.0000%
